# Exploring COVID-19–Related Stressors: Topic Modeling Study

**DOI:** 10.2196/37142

**Published:** 2022-07-13

**Authors:** Yue Tong Leung, Farzad Khalvati

**Affiliations:** 1 Department of Mechanical and Industrial Engineering University of Toronto Toronto, ON Canada; 2 Department of Diagnostic Imaging The Hospital for Sick Children Toronto, ON Canada; 3 Department of Medical Imaging University of Toronto Toronto, ON Canada; 4 Institute of Medical Science University of Toronto Toronto, ON Canada

**Keywords:** COVID-19, natural language processing, public health informatics, topic modeling

## Abstract

**Background:**

The COVID-19 pandemic has affected the lives of people globally for over 2 years. Changes in lifestyles due to the pandemic may cause psychosocial stressors for individuals and could lead to mental health problems. To provide high-quality mental health support, health care organizations need to identify COVID-19–specific stressors and monitor the trends in the prevalence of those stressors.

**Objective:**

This study aims to apply natural language processing (NLP) techniques to social media data to identify the psychosocial stressors during the COVID-19 pandemic and to analyze the trend in the prevalence of these stressors at different stages of the pandemic.

**Methods:**

We obtained a data set of 9266 Reddit posts from the subreddit \rCOVID19_support, from February 14, 2020, to July 19, 2021. We used the latent Dirichlet allocation (LDA) topic model to identify the topics that were mentioned on the subreddit and analyzed the trends in the prevalence of the topics. Lexicons were created for each of the topics and were used to identify the topics of each post. The prevalences of topics identified by the LDA and lexicon approaches were compared.

**Results:**

The LDA model identified 6 topics from the data set: (1) “fear of coronavirus,” (2) “problems related to social relationships,” (3) “mental health symptoms,” (4) “family problems,” (5) “educational and occupational problems,” and (6) “uncertainty on the development of pandemic.” According to the results, there was a significant decline in the number of posts about the “fear of coronavirus” after vaccine distribution started. This suggests that the distribution of vaccines may have reduced the perceived risks of coronavirus. The prevalence of discussions on the uncertainty about the pandemic did not decline with the increase in the vaccinated population. In April 2021, when the Delta variant became prevalent in the United States, there was a significant increase in the number of posts about the uncertainty of pandemic development but no obvious effects on the topic of fear of the coronavirus.

**Conclusions:**

We created a dashboard to visualize the trend in the prevalence of topics about COVID-19–related stressors being discussed on a social media platform (Reddit). Our results provide insights into the prevalence of pandemic-related stressors during different stages of the COVID-19 pandemic. The NLP techniques leveraged in this study could also be applied to analyze event-specific stressors in the future.

## Introduction

The COVID-19 pandemic has affected the lives of people globally for over 2 years. To mitigate infection, safety measures such as social distancing, lockdowns, and school closures have been implemented. The mental health of many has been impacted due to stress, anxiety, loneliness, and feelings of uncertainty about the pandemic [1]. Recognizing the common psychosocial stressors and their prevalence at different stages of the pandemic will allow health care providers and social workers to provide better mental health interventions and support. Several studies have been conducted to understand the COVID-19–related stressors and the impacts of COVID-19 on mental health [1-3]. The majority of the studies used questionnaires or interviews to obtain data. However, the process of obtaining data could be time-consuming and costly. As the pandemic is evolving rapidly, the prevalence of stressors may also be changing due to different factors such as the social distancing measures, coronavirus infection rates, and vaccine distribution. Survey methods may not be able to capture current COVID-19–related stress. In contrast, social media may allow close to real-time monitoring of the changes in stressors during the pandemic, as individuals actively share their feelings and difficulties on social media platforms. In this study, natural language processing (NLP) techniques are applied to identify COVID-19–related psychosocial stressors that are discussed on social media and to visualize the prevalence of stressors at different stages of the pandemic.

Social media platforms, such as Twitter and Reddit, are commonly used as the data source for obtaining insights regarding mental health status. As people share their feelings or experiences on the platforms, the content may reflect users’ emotions. The changes in emotions of the population could be reflected by their behavior on social media. Many researchers have utilized NLP techniques and social media data to analyze the mental health status of the population. De Choudhury et al [2] introduced a social media depression index to monitor the trends in depression across the population. Larsen et al [3] devised a system, called “We Feel,” that applies sentiment analysis and data visualization to tweets to obtain real-time insights into the emotional state of the population.

In the field of public health and informatics, some researchers have utilized NLP topic modeling to summarize COVID-19–related discussions on social media. Medford et al [4] observed the trend in topics in COVID-19–related tweets in the early stage of the outbreak. Jelodar et al [5] leveraged topic modeling to identify the topics that were being discussed on COVID-19–related subreddits such as \rCOVID19, \rCoronavirus, and r/CoronaVirus2019nCoV. Jang et al [6] used topic modeling to identify topics on Twitter to understand the reactions and concerns about COVID-19 in North America. Besides summarizing pandemic-related discussions, some research has leveraged NLP techniques on social media data to analyze the mental health impacts of COVID-19. Biester et al [7] observed changes in Reddit mental health support groups after the COVID-19 outbreak and obtained insights into the impact on mental health. In that study, the topic model was applied to identify topics such as family and school in the subreddits; then, time series analysis was leveraged to find which of the topics were affected after the outbreak of the pandemic. Low et al [8] used NLP techniques to characterize the differences in Reddit mental health support groups in the prepandemic and mid-pandemic periods.

Although some prior NLP research aimed at devising methods to utilize social media data to measure the population’s mental health status, few studies focused on identifying psychosocial stressors. According to the American Psychological Association [9], a psychosocial stressor is defined as “a life situation that creates an unusual or intense level of stress that may contribute to the development or aggravation of mental disorder, illness, or maladaptive behavior.” Mowery et al [10] developed an annotation scheme to identify depression symptoms and psychosocial stressors, such as problems with expected life course and problems with the primary support group, mentioned in tweets. Regarding the stressors during the pandemic, existing research used traditional methods such as questionnaires. Park et al [11] developed a questionnaire-based assessment tool for COVID-19–related stressors. The questions included whether individuals experienced the following in the past week: “risk of becoming infected,” “risk of unintentionally infecting other people,” “cancellation of meaningful personal or religious rituals,” and “loss of current job security or income.” People experiencing those stressors may also share their experiences on social media. If we could identify the stressors that were mentioned in posts, we would have real-time monitoring of changes. Our study aimed to provide an alternative method using NLP and social media data to obtain related data and summarize the prevalence of COVID-19–related stressors.

In this paper, we utilized the latent Dirichlet allocation (LDA) topic model to identify pandemic-related distress, by identifying the topics being discussed on the subreddit \rCOVID19_support. After applying the LDA model, we visualized the trends in the prevalence of topics at different stages of the pandemic. Several existing works leveraged NLP to analyze the population’s mental health status during the pandemic; however, existing studies did not explore the possibility of analyzing COVID-19–related stressors. This study focused on monitoring the stressors during the pandemic. Although most existing research focused on the mental health impacts at the beginning of the outbreak of the pandemic, the data set extracted in this study covered the posts on the subreddit starting from February 2020, the outbreak of the pandemic, up to July 2021. This allowed us to visualize the changes in the prevalence of stressors at different stages of the pandemic. Observing the trends can provide insights into the latest predominant stressors, and the findings could also be useful for mental health support providers and policy makers. We believe applying NLP to summarize text can help us obtain insights into mental health status and stressors during the pandemic.

## Methods

### Study Design

**Figure 1:**
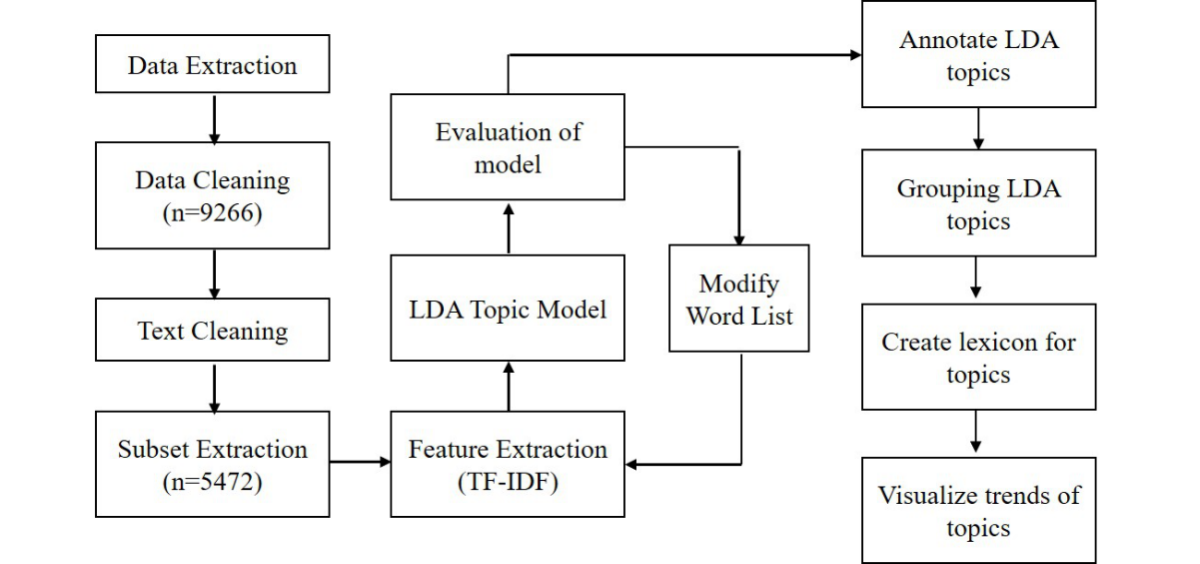
Overview of the research framework. LDA: latent Dirichlet allocation; TF-IDF: term frequency-inverse document frequency.

Figure 1 describes the overview of the study. First, we extracted the Reddit posts on \rCOVID19_support and preprocessed the data, including data cleaning and text cleaning. We then extracted a subset of posts that were likely to include content related to our research topic. Next, we created the LDA topic model to cluster the posts into different LDA topics for identifying the topics that existed in our data set and grouped the LDA topics with similar content into topic groups. By reviewing the keywords and example posts in those topic groups, we gained insights into the popular topics that existed in the data set. Once we identified the popular topics in our data set, we observed the frequently appearing words in each of the topics and identified the keywords for the topics. Next, we created psychosocial stressor lexicons, which were sets of keywords for each of the topics about the stressors. We then used the LDA topic model and the lexicons to label the topics that were covered in the posts. With the labels, we measured the number of posts with those topics for each month within our study period. Then, we could visualize the trends in the prevalence of topics on the subreddit.

### Data Collection and Preprocessing

In this study, Reddit was selected as the data source for applying machine learning models to obtain insights into the prevalence of stressors during COVID-19. There are several advantages of using Reddit in this study. De Choudhury and De [12] found that the anonymity of throwaway accounts on forums like Reddit can promote self-disclosure about mental health issues. Naseem et al [13] found that COVID-19–related discussions on Twitter focused on news and opinions about government policies, while the discussions on Reddit focused on how the pandemic affected people’s lives. As a result of these tendencies, Reddit has a high potential to be a better data source for identifying psychosocial stressors during the pandemic.

There are different subreddits related to COVID-19. We selected \rCOVID19_support for studying COVID-19-related stressors. Users on the platform \rCOVID19_support ask questions about COVID-19 and share their experience during hard times in the pandemic. The discussions on other subreddits, such as \rCOVID-19, \rCoronavirus, and \rCoronaVirus2019nCov focused on news and information instead of sharing experiences during the pandemic. As the topics discussed on \rCOVID19_support are more related to our research topic, we selected this subreddit as our data source.

Reddit posts were extracted from the subreddit \rCOVID19_support using the Pushshift API [14]. The data set included 9266 posts from February 14, 2020, to July 19, 2021. The posts marked as “[removed]” or “[deleted]” were excluded from the data set. After data cleaning, we applied the following preprocessing steps to avoid the noise influencing the topic model: First, we joined the texts in the titles and content of the posts; second, we removed the hyperlinks in the posts by removing tokens that included characters “www”; third, we replaced words spelled informally with the formal spelling, for example “ive” to “I have” and “ppl” to “people”; fourth, we lemmatized the words; fifth, we removed stop words, such as “would,” “they,” and ”are” in the text; sixth, we removed punctuation and numbers.

On Reddit, some of the posts are tagged by “flairs,” which describe the content or the nature of the posts. The flairs in the data set included: “Support,” “Questions,” “Discussions,” “Trigger Warning,” “Good News,” “Firsthand Account,” “Resources,” “Vaccines are SAFE,” “News,” “Biosafety Request,” “The answer is NO,” “Misinformation-debunked,” and “Desperate mod.”

Posts that are tagged with flairs such as “Resources” and “News” have a low tendency to include content related to psychosocial stressors. With the use of flairs tagged to the posts, we filtered posts with a low chance of including content related to our research topic. On the subreddit, some of the posts included content related to sharing information such as the latest news about COVID-19, potential adverse effects of vaccines, and tips about infection prevention. Some users asked questions such as which vaccines are safe, whether the adverse effects of vaccines are normal, and whether it is safe to visit their grandparents during the pandemic. The posts labelled with flairs “Support” and “Trigger Warning” have a high tendency to include content about users’ personal experiences, stressors, or feelings during the pandemic. Posts with flairs “News” and “Questions” tend to not include content about stressors. As we were focused on understanding stressors in this study, we extracted a subset to include posts that were labelled with the flairs that have a high tendency to include relevant content.

In the data set, 4654 posts were labelled by flairs, and 4612 posts were not. The number of posts tagged by each of the flairs is shown in Table 1. Missing flairs were predicted using a logistic regression model that was trained by the labelled data. Before training the classifier, the texts in the posts were represented by the term frequency-inverse document frequency (TF-IDF), which quantifies the significance of a given term compared with the other terms within the given document and within the given corpus [15]. The features used to train the classifier included LDA features (n=10), TF-IDF features (n=200), and 1 feature to describe whether the posts included hyperlinks (n=1). After filling in the missing flairs in the data set, 5472 data points were labelled by flairs that likely described stressors during COVID-19. These data points were extracted as a subset for analysis.

**Table 1:** Number of posts tagged by each of the flairs in the data set.

Flairs			Subset with labelled flairs (n=4654)		Subset with predicted flairs (n=4612)		Data set with labelled or predicted flairs (n=9266)
**Mental health support**							
	Support	2386		—^a^		—	
	Trigger warning	197		—		—	
	Deperate mod^b^	1		—		—	
	Total	2584		2888		5472	
**Discussion and questions**							
	Questions	1069		—		—	
	Discussion	597		—		—	
	Vaccines are SAFE	55		—		—	
	The answer is NO	7		—		—	
	Biosafety request	14		—		—	
	Total	1742		1417		3159	
**News and resources**							
	Good news	146		—		—	
	Resources	59		—		—	
	News	18		—		—	
	Misinformation—debunked	3		—		—	
	Total	226		225		451	
**Experience**							
	Firsthand account	102		—		—	
	Total	102		82		184	

^a^Not applicable.

^b^The flair in the original post was “Deperate mod,” which is likely a misspelling of “desperate mood.”

### Topic Model Training and Evaluation

In this study, we used the LDA topic model to identify the COVID-19–related distress that was mentioned on Reddit. The topic model is an unsupervised machine learning model that can be applied to different research topics such as computational social science and understanding scientific publications [16]. LDA is a topic model that is commonly used to summarize the topics on social media. The LDA model is based on the following assumptions: A topic is a combination of terms with a probability distribution, and a document is generated by a combination of topics with a probability distribution [16]. The algorithm starts by setting topic assignments randomly and then computes the distribution of words in a topic and the distribution of topics in a document. Then, the topic allocation of words is updated iteratively until convergence. The LDA model returns the distribution of topics in each of the documents and the distribution of words in each of the topics. With the topic allocation of documents, the dominant topic of each of the posts in our data set could be determined.

In this study, the LDA model in the Python scikit-learn package was used to identify the topics in the data set. The model was trained by TF-IDF features that were created from the data set. For each of the data points, the topic model outputs the proportions of contents belonging to each of the topics. The dominant topic for each of the posts was then identified by finding the topic with the highest value in the LDA output. The number of topics is the major hyperparameter of the LDA model, which affects the interpretability of topics. There are different ways to determine a suitable number of topics to achieve high interpretability of topics. In the study by Jelodar et al [5], the number of topics was first set to a high number then clustered into 18 topics by human judgement. In our study, we used a similar method to select the number of topics. First, we set the number of LDA topics to 100 and checked the dominant topic of each of the posts and found that only 25 LDA topics had more than 10 posts with corresponding topics. Then, we analyzed each of the 25 LDA topics and clustered them into 6 topics using human judgment. For each of the LDA topics, 3 sample posts with the highest LDA output percentage and 3 random posts with corresponding dominant topics were selected for manual review. Topic interpretability was manually evaluated by reviewing the data points selected. If the sample data points of the same dominant topics had similar contents, the topic could be named. After naming the LDA topics, some topics shared similar content with other topics. The LDA topics were then grouped into “topic groups.” We identified 6 topic groups: educational and occupational problems, family problems, fear of coronavirus, mental health symptoms, problems related to social relationships, and uncertainty about the development of the pandemic. The UMass coherence score of the LDA topic model is –2.106.

### Feature Engineering

The output of topic modeling is highly dependent on the feature vector (a matrix of TF-IDF values for each of the documents). For the first trial, the feature vector used to train the LDA model included TF-IDF with max_feature 300. This means the feature vector includes 300 columns of tokens, which are the terms consisting of one or more words that have the highest TF-IDF values in the given corpus. Then, the model was evaluated by the aforementioned methods, namely selecting sample posts from each of the topics and evaluating the topic coherence manually. In addition to evaluating LDA topic coherence, features were manually evaluated to determine whether they were likely to cause the LDA topic model to cluster posts in desired ways. For example, the topic model may group posts with tokens “suggestion,” “anyone,” and “thank you” into the same topic because those words tend to appear together when authors were asking for suggestions at the end of posts. However, clustering this topic does not help with understanding the stressors and may act as noise. To avoid this, those tokens were removed from the feature vectors. Besides removing those tokens, we also identified some tokens that were useful for identifying the topics. For example, the tokens “grocery shopping,” “maskless,” and “no mask” commonly appeared when users were expressing their fears of getting infected. We hypothesized that including those tokens in the feature vector would help the LDA topic model identify topics related to fear of coronavirus. The feature vector was then updated by selecting these tokens. Then, the LDA was trained by the new feature vector, leading the output of the model to be closer to the desired result. The iteration process to improve the topic model is illustrated in Figure 1. The iteration ended when the sample posts shared similar content within the same LDA topic. Using this iterative approach to feature selection and evaluation of the topic model output, the performance of the topic model was optimized.

### Psychosocial Stressor Lexicon

A psychosocial stressor lexicon is a list of keywords for each of the stressors. After the topic groups were defined, each of the topic groups was evaluated to search keywords that directly indicate the existence of topics in the text. For example, if the posts include education-related words such as “college” and “online learning,” it can be concluded that the authors have mentioned educational problems in the post. Lexicons were created for some of the topics. If a post included any of the words listed in the lexicons, it was assumed that the post included content related to the corresponding topics. Each of the posts could contain more than one topic. With the use of lexicons, each of the posts was annotated by whether it included content of each of the topics.

In LDA, topics are defined as a mixture of terms with different probability distributions; this means a word could belong to more than one topic and it could cause inaccuracy in the prediction. In contrast, the lexical approach has higher interpretability on topic classification, but it requires careful selection of keywords to avoid including terms belonging to more than one topic. In this study, we assumed that we do not have prior knowledge of how Reddit users expressed their feelings on the subreddit. To obtain insights into what topics existed in the subreddit and the keywords for each of the topics, we applied the LDA model before applying the lexical approach. In this study, the lexical approach was created for 2 purposes: first, to further analyze the subtopics within the topic group. Some of the topics may include some common words and have to be grouped into the same topic group in the LDA model. The topics in the same topic group could be separated by choosing unique keywords. The second reason for creating the lexicon was to verify the result from the LDA.

### Visualization

In previous steps, each of the data points was annotated with the LDA output, which represents the proportion of the content belonging to each of the topic groups, and the results of the lexical approach, which represent whether the post includes words that were listed in lexicons. Then, the monthly sum of the LDA model output for each topic groups was computed. The trend of topics in the data set can then be visualized and compared with the development of the pandemic. Regarding the pandemic development, the numbers of total cases, new cases per day, and vaccinated population were obtained from Our World Data [17]. In this study, only the numbers from the United States, the United Kingdom, and Canada are included because the majority of Reddit users are from these countries [18].

### Ethical Considerations

This study uses public datasets derived from social media and does not involve any human participants. As such it does not require ethics board approval.

## Results

### Topics Identified by the Topic Model

**Figure 2:**
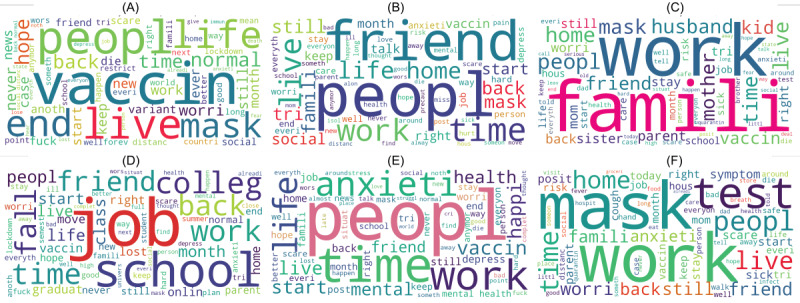
Word clouds for each of the topics identified: (A) uncertainty about the development of the pandemic, (B) problems related to social relationships, (C) family problems, (D) educational and occupational problems, (E) mental health symptoms, (F) fear of the coronavirus.

After grouping LDA topics, 6 topics were identified: (1) fear of coronavirus, (2) educational and occupational problems, (3) family problems, (4) problems related to social relationships, (5) mental health symptoms, and (6) uncertainty about the development of the pandemic. We grouped the posts according to the topics and created word clouds for each of the 6 subsets. Figure 2 shows the word clouds, which describe the frequently used words for each of the topics. The font size in the word clouds represents the frequency of words in the topics. By visualizing the keywords, the word clouds summarized the content for each of the topics.

We used t-distributed Stochastic Neighbor Embedding (t-SNE), which can be used to visualize high-dimensional data, to visualize the coherence of topic groups.

**Figure 3:**
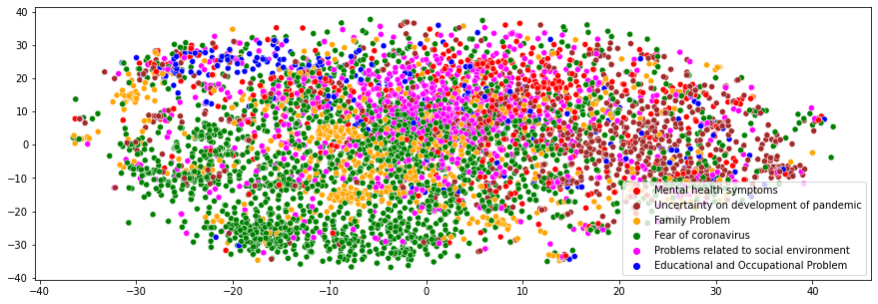
t-distributed Stochastic Neighbor Embedding (t-SNE) plot for the topic groups identified.

Figure 3 shows the t-SNE that describes the clustering of topic groups in our data. As the posts could include multiple topics, we could observe visible crossover between the topic clusters in the t-SNE. It shows the topics “fear of coronavirus” and “family problem” are similar. This could be because many posts related to family problems are about the worries of infecting family members and worries about the situations of parents who were infected.

The major topic in the data set was “fear of coronavirus.” The words frequently appearing in the topic of “fear of coronavirus” are shown in Figure 2. The words “home,” “live,” and “family” appeared frequently when the authors of posts explained they were living with family members, followed by expressing worries of infecting or getting infected by the family members. The words “mask,” “risk,” and “scare” appeared when the authors shared their worries about getting infected outside of their home.

The LDA model identified the topic of “educational and occupational problems.” Some students shared their feelings about the online learning experience, and some felt upset because they missed the social engagement from school activities. Some people expressed worries about finding jobs during the pandemic. Other posts mentioned the situation of current university students having online classes for the final year and worried about finding a job after graduation. Due to the existence of this type of content and tokens related to education and work such as “online learning,” “graduation,” “university,” and “find jobs” appearing together, we grouped the educational and occupational LDA topics into the same group.

The topic “family problems” included content about several subtopics: the worry of elderly parents getting infected, worry about parents who were already infected, and the anger of having opposing views with family members due to different acceptance of social distancing measures. The topic “mental health symptoms” included mentioning symptoms such as insomnia, obsessive compulsive disorder, and feeling depressed. Some of the users described their feelings and mental health symptoms on Reddit and asked for mental health support but did not explain the stressors. The topic “problems related to social relationships” included content about loneliness and uncertainty about the balance between social activities and pandemic safety measures. The topic “uncertainty on development of pandemic” refers to the content about the worry of the pandemic lasting forever. For this topic, some posts included discussions about whether vaccination would help get life back to normal.

### Trends in LDA Topic Groups

**Figure 4:**
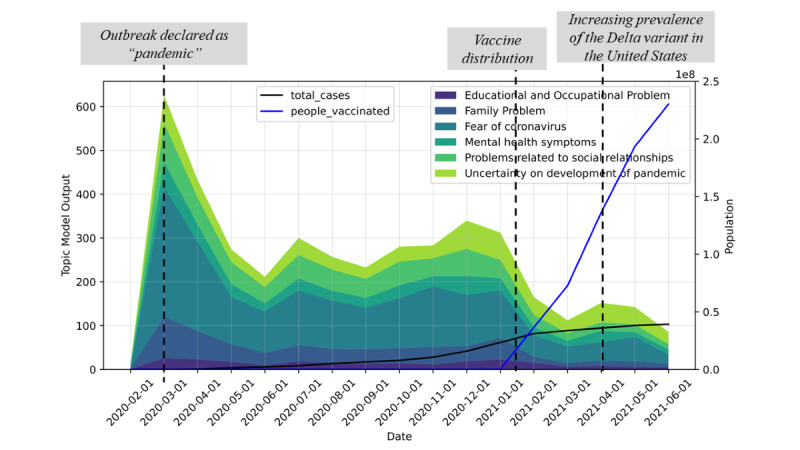
Trend of topics on \rCOVID19_support. The stacked area plot represents the sum of the latent Dirichlet allocation (LDA) output for each month, for each of the topics. The line plot represents the total number of cases and the vaccinated population.

On March 11, 2020, the World Health Organization declared COVID-19 a “pandemic” [19]. As shown in Figure 4, the number of posts on the subreddit was the highest in March 2020. The number declined from April 2020 to June 2020, then rose steadily from September 2020 to December 2020. The number of posts related to stressors then declined in February 2021, and vaccine distribution started between January 2021 and February 2021.

**Figure 5:**
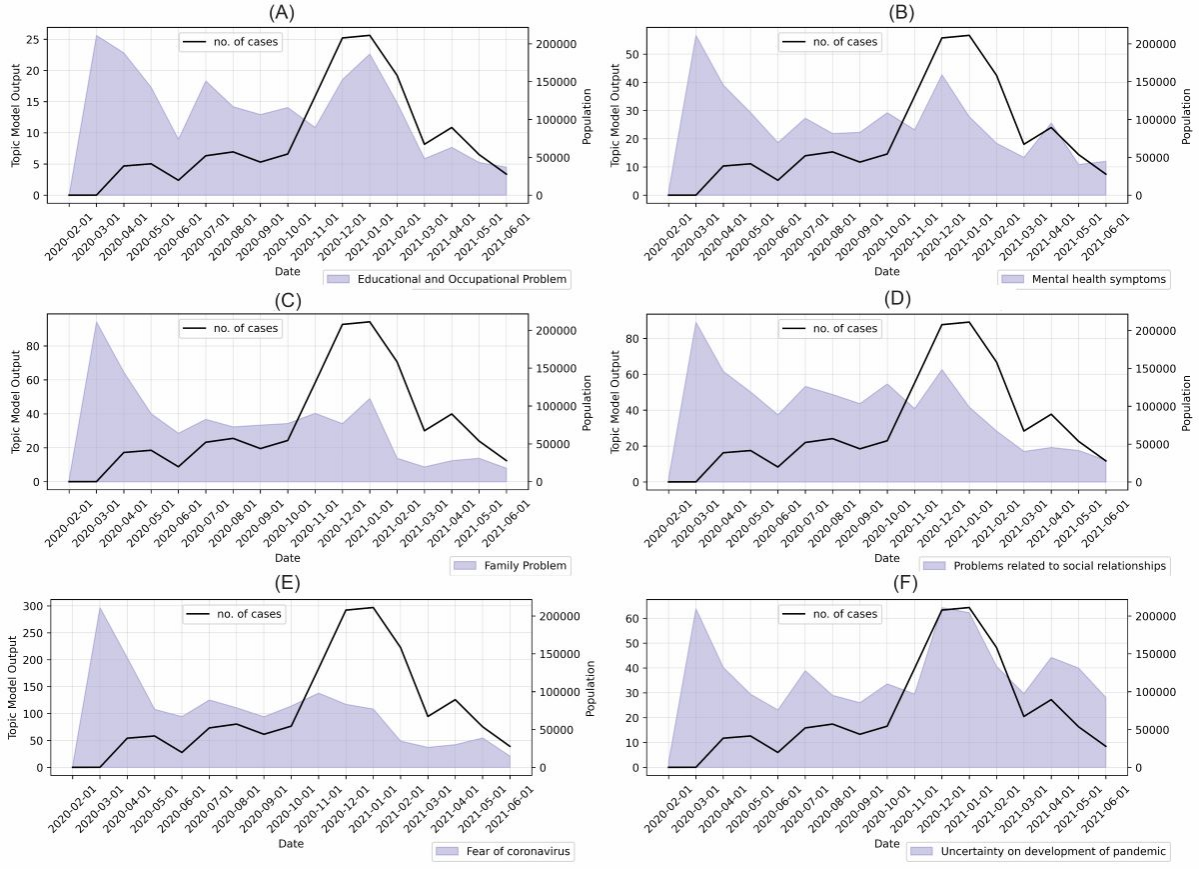
Trends in the prevalence of topic groups: (A) educational and occupational problems, (B) mental health symptoms, (C) family problems, (D) problems related to social relationships, (E) fear of the coronavirus, and (F) uncertainty on the development of the pandemic. The line plots represent the number of cases for each month. The area plots represent the prevalence of topics, which was measured using the output of the latent Dirichlet allocation (LDA).

The trends in the prevalence of topics have been plotted separately in Figure 5. To understand the relationship between the trends in stressors and the number of COVID-19 cases, the daily number of cases was also included.

As shown in Figure 5, the number of posts mentioning the fear of coronavirus did not significantly change from May 2020 to December 2020, although the daily number of cases had a significant increase in November 2020. This suggests the fear of coronavirus was independent of the actual number of cases. The number of posts with the topic of “fear of coronavirus” was the highest in March 2020, when COVID-19 was first declared a “pandemic.” In February 2021, the discussions about the fear of coronavirus significantly reduced. This suggests the distribution of vaccines may have reduced the perceived risks of infection with the coronavirus.

The prevalence of the topics “family problems” and “educational and occupational problems” dropped in February 2021. For the topic of uncertainty, its prevalence declined in February 2021 but increased in April 2021, when the new variant was becoming prevalent in the United States. The most common topic in December 2020 and January 2021 was “uncertainty about development of pandemic.” Starting from May 2020, the prevalence had a high correlation with the number of cases.

**Figure 6:**
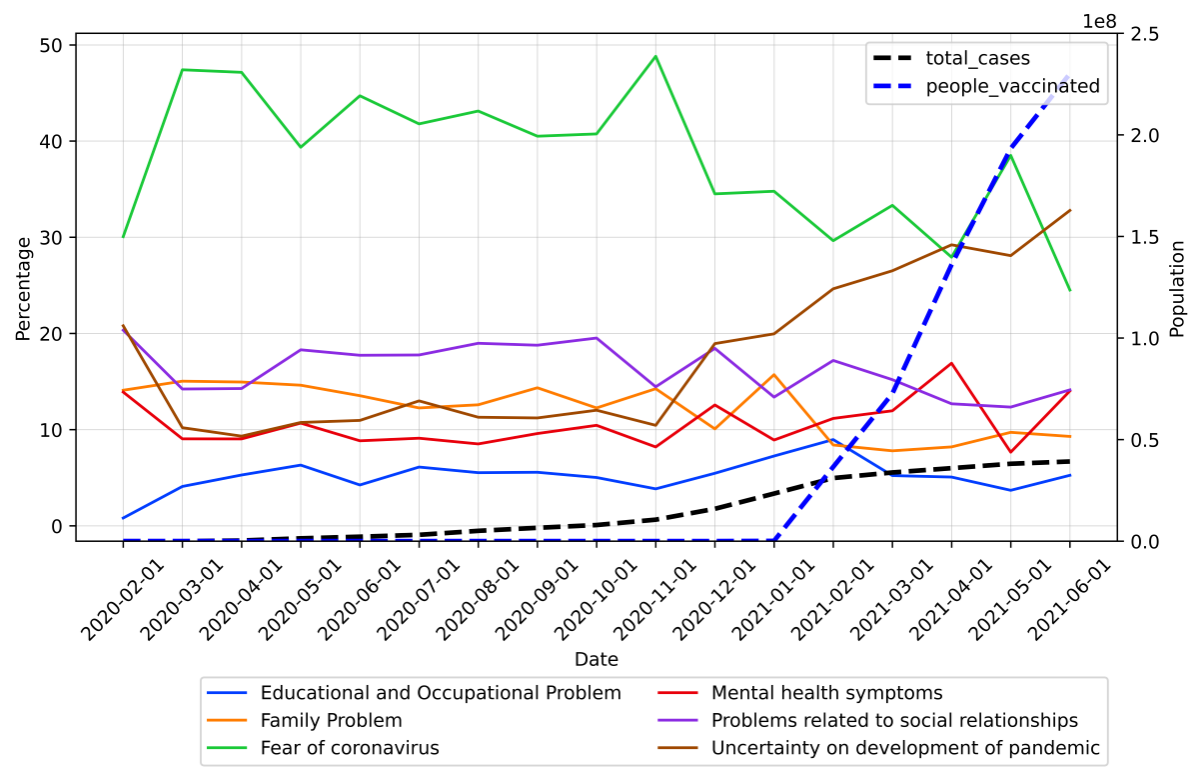
Trend on proportions of topics mentioned on \rCOVID19_support. The dashed lines represent the total number of cases and the vaccinated population. The solid lines represent the proportion of each topic for each month.

To determine the distribution of topics in each month, we calculated the percentage of posts belonging to each topic in each month, and the corresponding trend was plotted (Figure 6).

Figure 6 displays the proportion of each topic with respect to all topics. As shown, “Fear of coronavirus” was the major topic of discussion on the platform. From March 2020 to November 2020, more than 40% of the content on the forum belonged to the fear of coronavirus topic. Starting from December 2020, the proportion was below 40% and showed a decreasing trend, while the proportion of posts about “Uncertainty on development of pandemic” steadily increased. In June 2020, the proportion of the topics about pandemic development was higher than the topics about fear. For other topics, the prevalence did not significantly change. The results suggest that the major stressor related to COVID-19 shifted from the fear of getting infected to a feeling of uncertainty about the development of the pandemic.

### Lexicon Approach

Table 2 shows the lexicon created based on the result of the LDA model. The lexicons were created for 2 purposes: first, to further analyze the subtopics within the topic group. As explained in previous sections, the LDA topics of occupational problems and educational problems had to be grouped. By reviewing the word clouds and sample texts of the topic group, we listed the words that could identify the educational and occupational problems separately. Therefore, we could analyze the prevalence of each of these subtopics. The second reason for creating the lexicon was to verify the result from the LDA. Lexicons about coronavirus and pandemic development have been created to verify the results from the LDA. For example, according to the randomly selected sample posts in each topic group, posts that include the tokens “no mask” and “without mask” mentioned the worries of being infected by people who do not wear masks on the street. Since the topic annotations with the lexicon approach are more explainable, it was used to verify the LDA. After annotating the topics, the trend on the prevalence of topics with the LDA approach and lexicon approach were visualized for comparison.

**Figure 7:**
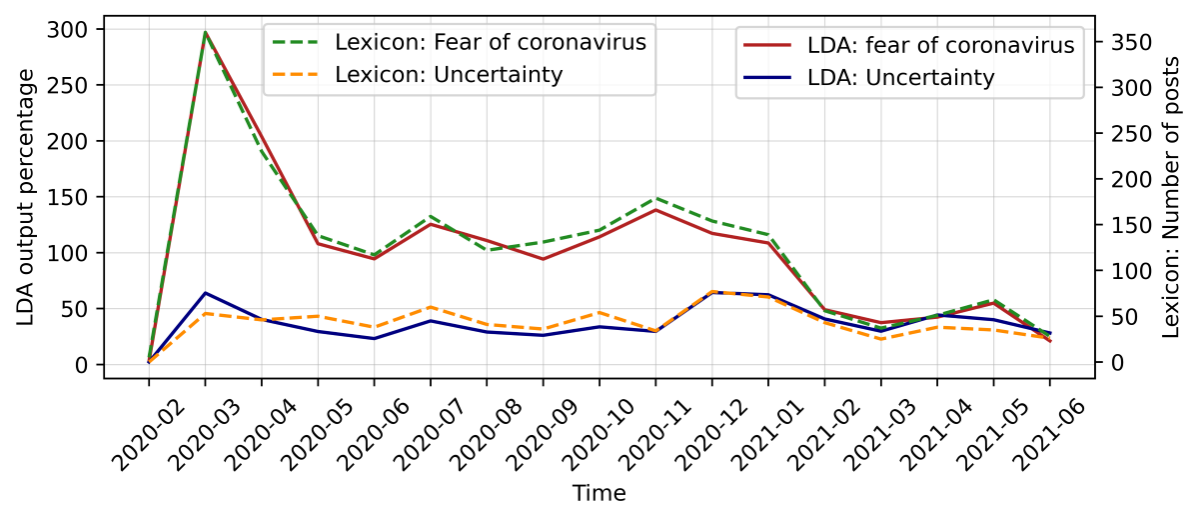
This figure compares the trends in the prevalence of topics between the latent Dirichlet allocation (LDA) model (solid lines) and the lexicon (dashed lines).

According to Figure 7, the results using the 2 approaches were comparable. The Person correlation coefficient of the results for the topic “Fear of coronavirus” from the 2 approaches was 0.995, and the correlation coefficient for the “Pandemic development” topic was 0.829.

The keywords for each of the topics were selected by evaluating the sample posts from the topic groups in the LDA model. As we assumed we had no prior knowledge of which mental health issues were expressed on the platform and the commonly used keywords for each of the topics, the LDA model was applied before using the lexical approach. Once the keywords and topics were obtained, we could use the lexical approach to label the topics mentioned in each of the posts and visualize the trends. In some cases, the posts may describe the topics without using the keywords. For example, in the topic “mental health symptoms” in the LDA model, the text in a post may include the words “feel,” “anxious,” “depressed,” and “tired”; however, those words are also common in other topics. Therefore, it may be unsuitable to use those keywords to identify the topic “mental health symptoms” using the lexical approach. For this case, the LDA model is needed to identify that topic. In this study, both methods were used, showing similar results for the topics “Fear of coronavirus” and “Pandemic development” (measured using the correlation coefficient).

We also plotted the trends in the number of posts for each topic separately in Table 2. The keywords for each of the topic groups were obtained by evaluating the sample posts. If a post contained words included in the lexicon, the post was assumed to include the content of the corresponding topic. Each post could include content on more than 1 topic. To observe relationships with the development of the pandemic, the total number of COVID-19 cases and the vaccinated population size were included in the same figure.

**Figure 8:**
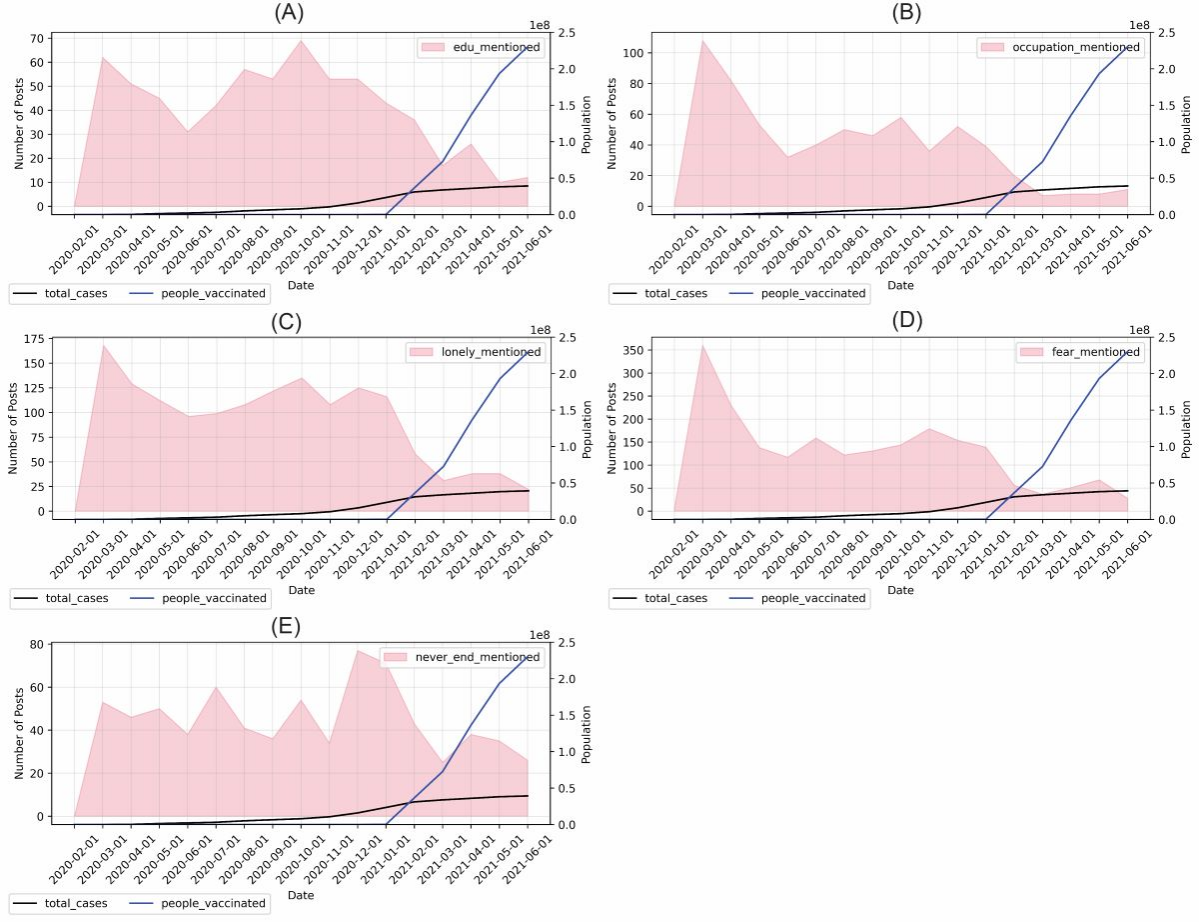
Trend on the number of posts mentioning each of the topics in the lexical approach: (A) educational problems, (B) occupational problems, (C) lonely, (D) fear of coronavirus, (E) pandemic development.

Figure 8 shows the result of the lexical approach for each of the topics. According to Figure 8, the number of posts mentioning “education problems” had a declining trend from March 2020 to June 2020. The peak, in March 2020, could be due to the start of school closures, when both students and teachers were not familiar with online learning. After that, the students may have adapted to the situation. The number of posts with content regarding online learning may have declined when the summer holidays approached. However, the number of posts with the topic of “educational problems” increased from June 2020 to October 2020. This may suggest that the prevalence of education-related stress increased with time. Then, the number of related posts decreased, starting from October 2020. Compared with other topics, the prevalence of education-related discussions started decreasing before the vaccine distribution. The trend was independent of the number of cases.

To understand which stressor was the most prevalent at different stages of the pandemic, we measured the percentage of posts containing the topics in each month and plotted the trend.

**Figure 9:**
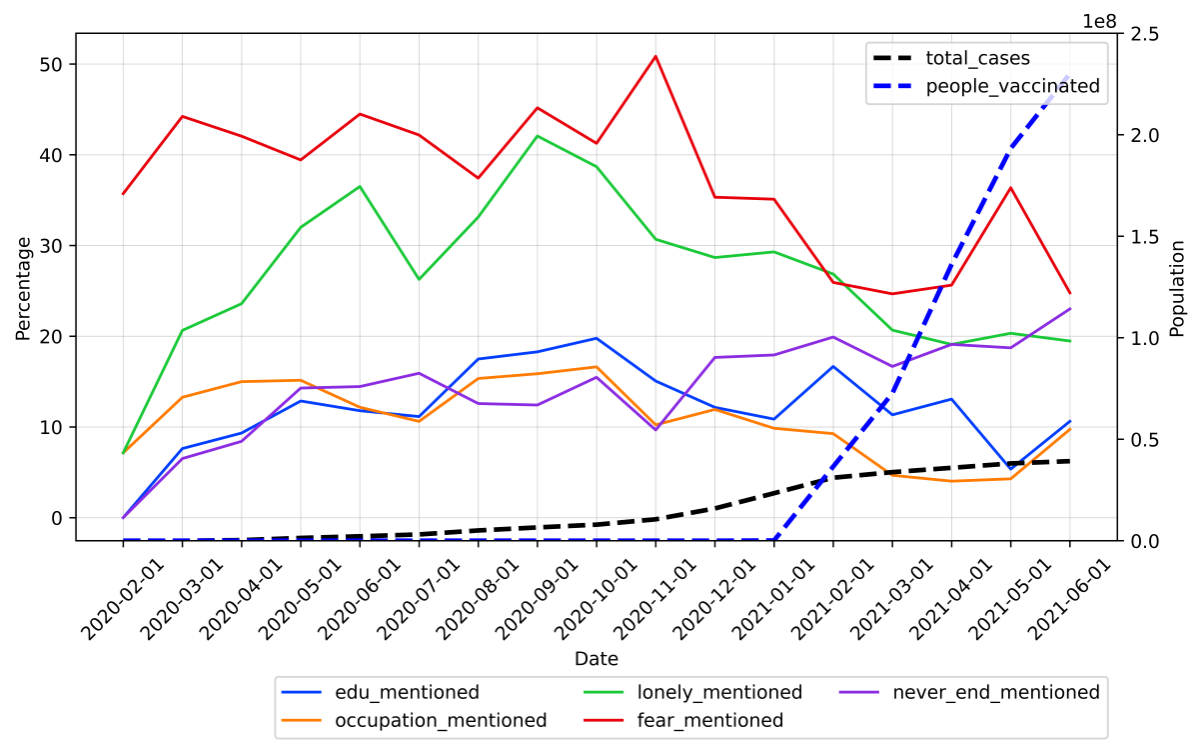
Percentage of posts mentioning each of the topics in each month (dominant topic).

According to Figure 9, in September 2020, more than 40% of the posts mentioned loneliness. After September 2020, there was a decreasing trend for the topic of loneliness.

**Table 2:** Lexicon for COVID-19 stressors.

Topics	Tokens
Education problems	college, online learning, class, semester, freshman
Occupational problems	lost job, unemployed, laid off, income, money, quit job, career
Lonely	social interaction, interact, connection, lonely, friendless, feel alone, loneliness, friendless, social life, friendship, socialize, make friends, new friends, disconnected
Fear of coronavirus	no mask, without mask, maskless, unmasked, grocery, panic, precautions, coworker, cough, exposed, wash, temperature, OCD
Pandemic development	forever, permanent, back normal, new normal, ever end, never ending, endless, lose hope, normal life

## Discussion

### Principal Findings

In this study, with the use of the topic model on Reddit data (subreddit \rCOVID19_support), we identified 6 topics related to the pandemic, which were “fear of coronavirus,” “educational and occupational problems,” “family problems,” “problems related to social relationships,” “mental health symptoms,” and “uncertainty about development of pandemic.”

According to the result of our study, the prevalence of discussions on “fear of coronavirus” dropped significantly after the start of vaccination. One of the possible explanations is that the increase in the vaccinated population may have reduced the perceived risk of COVID-19. Perez-Arce et al [20] suggested the uptake of vaccines has potential benefits on mental health because it reduces the perceived risks of infection; people who are recently vaccinated are less worried about being infected and would engage in social activities that they considered as conveying a high risk of infection before. In the study, it was also suggested that the unvaccinated population could be less worried about being infected or infecting family members as more people became vaccinated. This could explain the significant decrease in the prevalence of discussions on “fear of coronavirus” and also the topics related to family problems. In the LDA topic group about family, there were posts related to the worries of infecting family members, struggles about whether one should avoid visiting grandparents, and arguments with family members due to different opinions about the balance between social distancing and social activities. These stressors may have been alleviated by the decrease in perceived risks of infection when the vaccinated population was increasing.

The number of posts about “uncertainty about pandemic development” did not have a notable drop while the vaccinated population was growing, but the trend shows a correlation with the number of cases. People may have been uncertain about the length of lockdown and how long social distancing requirements would last. This could be correlated with the number of cases instead of the perceived risks of infections. Briscese et al [21] suggested that, when the length of social distancing measures is extended, the population may think the goal is unachievable and feel frustrated. In our study, the prevalence of discussions about uncertainty significantly rose in April 2021, when the Delta variant was becoming prevalent in the United States. This trend may reflect the frustration of knowing the length of social distancing measures would need to be extended due to the new variant.

### Comparison With Prior Research

In our study, the LDA models identified some of the COVID-19–related stressors proposed in prior studies. Taylor et al [22] devised the “COVID Stress Scale” to measure COVID-19–related distress and identify people in need of mental health services. The scale includes symptoms such as “danger and contamination fears” and “compulsive checking and reassurance seeking.” The study found that some people may have worries such as “social distancing is not enough to keep me safe from the virus,” “people around me will infect me with the virus,” and “can’t keep my family safe from the virus.” In our data set, those worries were expressed in the LDA topic group “fear of coronavirus.” In the research by Park et al [11], the most common COVID-19–related stressors in April 2020 were “reading/hearing about the severity and contagiousness of COVID-19” and “uncertainty about length of quarantine and social distancing requirements.” Those stressors were also identified by the LDA model applied to the \rCOVID19_support posts in our study. This verifies the potential of using this subreddit to capture the trend of major COVID-19–related stressors.

The trend observed in our study is consistent with those of prior studies. Yarrington et al [1] studied the changes in sentiment such as anxiety, tiredness, and depression during the different stages of the COVID-19 pandemic, with the use of data collected by a mental health app in the United States. The results of the study showed that the anxiety level was the highest in the pre stage (February 2, 2020 to March 11, 2020) and then decreased and remained stable during the acute stage (March 12 2020 to April 15, 2020) and sustained stage (April 16, 2020 to July 6, 2020) of stay-at-home orders. Daly and Robinson [23] assessed the psychological distress in the United States using the Patient Health Questionnaire-4 (PHQ-4). Distress levels also declined from April 2020 to June 2020. In our study, the number of posts mentioning the fear of coronavirus was the highest in March 2020, dropped in April 2020 and May 2020, and then remained stable from May 2020 to November 2020.

### Limitations

In this study, the prevalence of stressors was only compared with the number of cases and the vaccinated population and not compared with specific safety measures in specific cities. This was due to the anonymity on Reddit. The demographic information of users was unknown, and the data set obtained in this study included posts written by users in different countries. Every city has implemented social distancing measures and lockdowns at different times, depending on the number of cases and the hospitalization rates. Due to this situation, we could not analyze the relationship between mental health status and the safety measures.

Similar to other studies that utilized social media data, the data extracted could only represent the population who would share their experience and feelings on social media. During the pandemic, children and older adults were strongly impacted by the changes, but it is unlikely that they shared their experiences on Reddit or other social media. To understand the needs of people with different demographics, questionnaires or interview-based studies are still required.

### Implications for Public Health and Future Studies

According to the results of this study, the stressors that were caused by perceived risks were alleviated since the beginning of the vaccine distribution. However, the stressors related to the frustration of uncertainty on the length of social distancing measures then became the major stressors. Lockdown and social distancing policies may depend on the hospitalization rate, transmissivity, and severity of the virus. With a high proportion of the population vaccinated and more experience with handling COVID-19 patients, lockdowns such as those that occurred in the first 2 waves of COVID-19 are not likely to be required. In terms of alleviating mental distress, the government may consider explaining to the public that the health care system is prepared to handle new outbreaks of coronavirus and we are in the process of getting back to normal life.

With the use of the lexicons created in this study, we obtained the posts or the sentences that described the worries of getting infected and the uncertainty about whether the pandemic is never-ending. This could be used as a data set for training machine learning classifier models to detect tweets that describe the stressors. Unlike Reddit posts, geographic information is available for tweets. By specifying the users’ location of tweets, we could analyze the mental distress impacts of specific social distancing policies. We could also establish time series models to quantify and predict the expected effects on stressors. This could help policy makers to estimate the impacts on mental distress before implementing a policy.

In 2022, a new variant, Omicron, became prevalent, and there were updates to social distancing measures. In the future, we can use similar methods in this study to compare the trends in stressors at the time of the Delta variant and Omicron variant.

### Conclusions

In this study, we applied topic modeling to a data set that contained Reddit posts in \rCOVID19_support to identify the COVID-19–related psychosocial stressors and to visualize the trend in the prevalence of the stressors. Compared with existing research, which utilized NLP techniques on social media data to study the mental health impacts of the pandemic, our study focused on stressors instead of mental health status. The data set used in this study included posts that were created in a time period of more than 1 year during the pandemic. This allowed us to compare the difference in the prevalence of stressors before and after vaccines were distributed. The proposed topic model also allowed for monitoring the dominant stressors, which enables mental health support providers to notice changes in stressors at different stages of the pandemic. This study demonstrated the potential of using topic modeling on social media discussions to identify event-specific stressors and create a dashboard to analyze and monitor the trends. We hope the findings in this study will provide insights for health care providers and social workers to address the needs of COVID-19–related mental health support. Furthermore, we hope the NLP techniques used in this study will be applied to analyze psychosocial stressors and create corresponding lexicons of future events such as pandemics, protests, or financial crises.

## Abbreviations

LDA: latent Dirichlet allocation
NLP: natural language processing
PHQ-4: Patient Health Questionnaire 4
t-SNE: t-distributed Stochastic Neighbor Embedding
TF-IDF: term frequency-inverse document frequency
